# Inhibition of malignant thyroid carcinoma cell proliferation by Ras and galectin-3 inhibitors

**DOI:** 10.1038/cddiscovery.2015.47

**Published:** 2015-11-02

**Authors:** A Menachem, O Bodner, J Pastor, A Raz, Y Kloog

**Affiliations:** 1 Department of Neurobiology, The George S. Wise Faculty of Life Sciences, Tel Aviv University, Tel Aviv, Israel; 2 The Departments of Oncology and Pathology, School of Medicine, The Karmanos Cancer Institute, Wayne State University, Detroit, MI, USA

## Abstract

Anaplastic Thyroid carcinoma is an extremely aggressive solid tumor that resists most treatments and is almost always fatal. Galectin-3 (Gal-3) is an important marker for thyroid carcinomas and a scaffold of the K-Ras protein. S-trans, transfarnesylthiosalicylic acid (FTS; Salirasib) is a Ras inhibitor that inhibits the active forms of Ras proteins. Modified citrus pectin (MCP) is a water-soluble citrus-fruit-derived polysaccharide fiber that specifically inhibits Gal-3. The aim of this study was to develop a novel drug combination designed to treat aggressive anaplastic thyroid carcinoma. Combined treatment with FTS and MCP inhibited anaplastic thyroid cells proliferation *in vitro* by inducing cell cycle arrest and increasing apoptosis rate. Immunoblot analysis revealed a significant decrease in Pan-Ras, K-Ras, Ras-GTP, p-ERK, p53, and Gal-3 expression levels and significant increase in p21 expression levels. In nude mice, treatment with FTS and MCP inhibited tumor growth. Levels of Gal-3, K-Ras-GTP, and p-ERK were significantly decreased. To conclude, our results suggest K-Ras and Gal-3 as potential targets in anaplastic thyroid tumors and herald a novel treatment for highly aggressive anaplastic thyroid carcinoma.

## Introduction

Thyroid cancer is the most frequent endocrine neoplasia and its incidence has been increasing in the past few decades. Most patients with thyroid cancer have a good outcome when treated with standard therapies, which are: surgery, chemotherapy, and radiotherapy.^1^ The prognosis for those with resistant or recurrent disease is poor. The classification of thyroid cancers is done by their histopathological and clinical characteristics, from well-differentiated to undifferentiated types.^2^ Well-differentiated types include papillary and follicular thyroid carcinoma. They are more common and have a good prognosis. Anaplastic thyroid carcinomas are undifferentiated, extremely aggressive, less common and usually lethal.^3^

Several important markers for thyroid carcinomas have been described. One of these is galectin-3 (Gal-3), which acts extracellularly as a *β*-galactoside-binding protein and intracellularly as a scaffold of the K-Ras protein.^4–6^ Galectins are often overexpressed in cancerous cells. In many situations, this altered galectins expression correlates with the aggressiveness of the tumor and the acquisition of the metastatic phenotype, indicating that galectins might modulate tumor progression and influence disease outcome.^7–11^

The galectins family might have an important role in the membrane anchorage of Ras and in Ras-mediated cell transformation.^5,12^ Ras proteins (H-Ras, K-Ras and N-Ras) are members of the GTPase family that have vital roles in cell signaling pathways regulating cell growth, differentiation, proliferation, and cell death.^13,14^ Ras mutations are known to be involved in approximately 25–30% of all human cancers.^15,16^ To cause malignant transformation, oncogenic Ras must associate with cellular membranes.^17,18^ It was found that Gal-3 binds to oncogenic Ras proteins, but preferentially to K-Ras, promotes the activation of important signaling cascades, including RAF1, phosphatidylinositol 3-kinase, Ras signaling pathway, and regulate gene expression at the transcriptional level.^5^

S-trans, transfarnesylthiosalicylic acid (FTS; Salirasib) is a synthetic small molecule that acts as a potent Ras inhibitor. FTS dislodges all types of oncogenic Ras proteins from their membrane anchorage sites and inhibits Ras transformation both *in vitro* and *in vivo*.^19–22^ In previous studies that were done in our lab, we found that FTS inhibits the growth of thyroid cancer cells (ARO) *in vivo* and *in vitro* and decreased K-Ras, K-Ras-GTP, p-ERK and Gal-3 levels.^23^ Modified citrus pectin (MCP) is a water-soluble citrus-fruit-derived polysaccharide fiber that specifically inhibits Gal-3. Pectin is a highly complex branched polysaccharide fiber rich in galactoside residues and present in all plant cell walls. In its naive form, citrus pectin (CP) has a limited solubility in water and is unable to interact with Gal-3, but its modified form (MCP) acts as ligand for Gal-3.^24^ MCP induces apoptosis in multiple myeloma cells resistant to conventional therapies.^25^ It also inhibits tumor growth, angiogenesis and spontaneous metastasis of breast and colon carcinoma cells in nude mice.^26^

Although standard therapies are effective for most patients with thyroid cancer, they do not work well on patients with aggressive anaplastic thyroid carcinomas and the prognosis for these patients is poor. In the current study, we examine the combined treatment of Ras inhibitor, FTS, and Gal-3 inhibitor, MCP on anaplastic thyroid carcinoma cells (ARO) *in vitro* and *in vivo*. Our results show that combined treatment with FTS and MCP inhibit tumor cells proliferation *in vitro* and tumor growth *in vivo*. In addition, FTS with MCP induced cell cycle arrest at the G1 phase and apoptosis. These novel findings can be clinically relevant and can lead to the development of new approaches for treating patients with aggressive anaplastic thyroid carcinomas.

## Results

### FTS and MCP work synergistically on ARO cells

**Figure 1:**
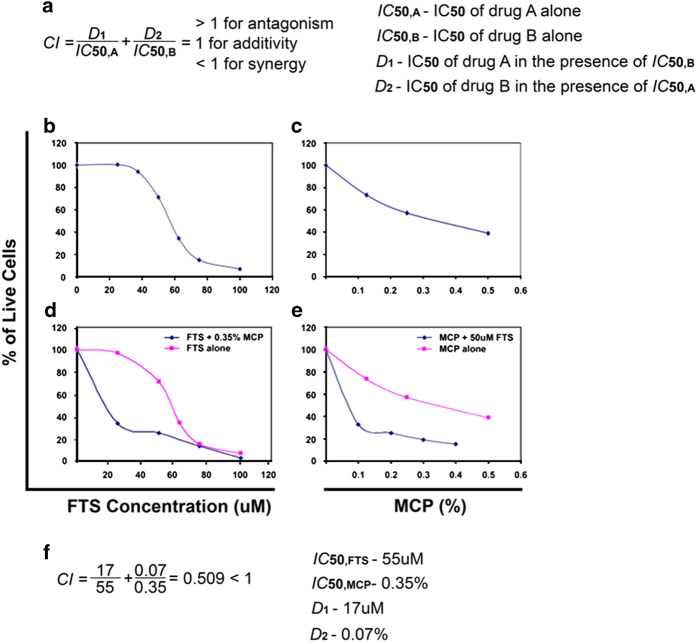
Combined treatment of FTS and MCP works synergistically on ARO cells. (**a**) The combination index (CI), proposed by Berenbaum. If the two drugs act synergistically, the CI will be lower than one as described in the results. (**b–e**) Results of cell viability tests. (**b**) FTS dose–response (*μ*M) with an IC_50_ of 55 *μ*M. (**c**) MCP dose–response (%) with an IC_50_ of 0.35%. (**d**) IC_50_ value of FTS in the presence of 0.35% MCP reduced the number of live cells with an IC_50_ of 17 *μ*M. (**e**) IC_50_ value of MCP in the presence of 50 *μ*M FTS reduced the number of live cells with an IC_50_ of 0.07%. (**f**) Our results show that combination of FTS and MCP has a CI value of 0.509, thus suggesting the drugs work synergistically in ARO.

To study the form of interaction between FTS and MCP, we used the combination index (CI), proposed by Berenbaum (Figure 1a).^27^ In practical application, we determine the half-maximal inhibitory concentration (IC_50_) of one drug in the presence of a constant concentration of the other. If the two drugs act synergistically, lower concentrations would be needed in the mixture to achieve the same effect, and the CI will be lower than one. We determined the IC_50_ value of each drug given alone and found that FTS and MCP IC_50_ values were 55 *μ*M and 0.35%, respectively (Figures 1b and c). Next, we determined the IC_50_ value of FTS in the presence of MCP’s IC_50_ concentration, and vice versa. We found that FTS reduced the number of live cells with an IC_50_ of 17 *μ*M and MCP reduced the number of live cells with an IC_50_ of 0.07% (Figures 1d and e). Our results show that mixture of FTS and MCP has a CI value of 0.5, and work synergistically on ARO cells (Figure 1f).

### Combined treatment with FTS and MCP downregulate active Ras-GTP p-ERK and Gal-3 expression

**Figure 2:**
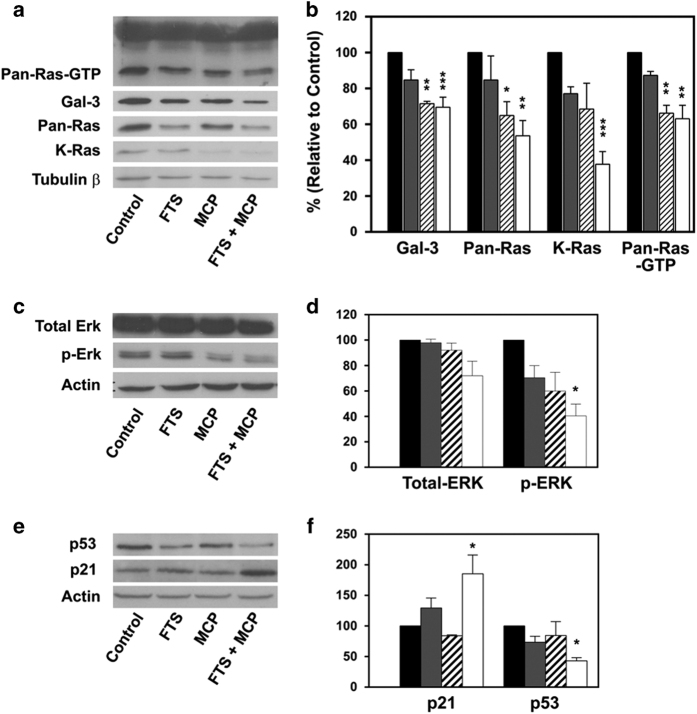
Combined treatment of FTS and MCP regulate Ras and its downstream signaling molecules. Combined treatment of FTS and MCP decreases the expression levels of, Galectin-3, Pan-Ras, K-Ras, Pan-Ras-GTP, p-ERK and p53, and increased the expression levels of p21. ARO cells were plated and treated with 75 *μ*M FTS and 0.35% MCP, as described in Methods and Materials. After 48/72 h, cells were lysed and subjected to immunoblotting. (**a**) Representative Immunoblots of Gal-3, Pan-Ras, and K-Ras normalized to *β*-tubulin, and Pan-Ras-GTP visualized by ECL. (**b**) Apparent levels of Galectin-3, Pan-Ras, K-Ras and Pan-Ras-GTP determined by densitometry (a.u.) of the immunoblots. Gal-3, Pan-Ras, K-Ras and Pan-Ras-GTP levels were significantly decreased in FTS+MCP-treated cells (white bar) compared with untreated cells (black bar), FTS-treated cells (gray bar) and MCP-treated cells (upward diagonal cells). Gal-3, Pan-Ras, K-Ras and Pan-Ras-GTP levels were significantly decreased in FTS+MCP-treated cells (**c**) Representative Immunoblots of total ERK and p-ERK normalized to actin, visualized by ECL (**d**) Apparent levels of total ERK and p-ERK determined by densitometry (a.u.) of the immunoblots. P-ERK levels were significantly decreased in FTS+MCP-treated cells (white bar) compared with untreated cells (black bar), FTS-treated cells (gray bar) and MCP-treated cells (upward diagonal cells). (**e**) Immunoblots for p21 and p53 normalized to actin. (**f**) Apparent levels of p21 and p53 determined by densitometry (a.u.). p21 levels were significantly increased and p53 levels were significantly decreased in FTS+MCP-treated cells (white bar) compared with untreated cells (black bar), FTS-treated cells (gray bar) and MCP-treated cells (upward diagonal cells). Results are presented as means±S.E.M.; one-way ANOVA analysis revealed significant differences between the groups; *post hoc* analysis was performed by the HSD test *P<0.05, **P<0.01, ***P<0.001.

To study the effect of combined treatment with FTS and MCP on protein expression, we treated ARO cells with FTS and MCP for 48 h and assessed protein expression by immunoblot analysis. Ras-GTP levels were assessed by GTPase pull-down assays, as described in Materials and Methods. As shown in Figures 2a and b, treatment with FTS+MCP significantly decreased Ras-GTP and total Ras levels by 37 and 46%, respectively (*P*<0.01, one-way ANOVA; *P*<0.01, Tukey's honest significant difference (HSD) test), and total K-Ras levels by 62% (*P*<0.001, one-way ANOVA; *P*<0.001, Tukey's HSD test) relative to untreated cells. To further assess the influence of combined treatment on Ras signaling pathways, we examined phospho-ERK (p-ERK), an important downstream protein in Raf/MEK/ERK pathway expression levels (Figure 2c). We found that although FTS+MCP treatment affected total ERK slightly (Figure 2d; *P*>0.05, one-way ANOVA), it significantly reduced p-ERK expression levels by 60% relative to untreated cells (Figure 2d; *P*<0.05, One-way ANOVA; *P*<0.05, Tukey's HSD test). As p-Akt is not detected in ARO cells, it was not used as readout for Ras signaling.^23^

Next, we examined the effect of FTS and MCP on Gal-3 levels. Although MCP and FTS+MCP significantly decreased Gal-3 expression levels to 70% relative to untreated cells, FTS had minor effect on Gal-3 expression levels (Figures 2a and b; *P*<0.01, one-way ANOVA; *P*<0.05, Tukey's HSD test).

### Combined treatment with FTS and MCP increase p21 and reduce p53 expression levels

Next, we examined whether combined treatment with FTS and MCP affect p21 and p53 expression (Figure 2e). P21 is an important cell cycle regulator, which induce cell cycle arrest at G1 and G2/M phases.^28^ In addition, previously it has been shown that active Ras negatively regulates p21 expression.^23^ We found that FTS+MCP significantly increased p21 levels by 85% relative to untreated cells (Figure 2f; *P*<0.01, one-way ANOVA; *P*<0.01, Tukey's HSD test). P53 is an important tumor suppressor gene, which positively regulates p21 transcription. Previously, it was shown that Ras inhibition leads to transcriptional activation of p53.^29,30^ We found that FTS+MCP significantly decreased p53 levels by 66% relative to untreated cells (Figure 2f; *P*<0.0001, one-way ANOVA; *P*<0.0001, Tukey's HSD test).

### Combined treatment with FTS and MCP induces G1 cell cycle arrest and apoptosis in ARO cells

**Figure 3:**
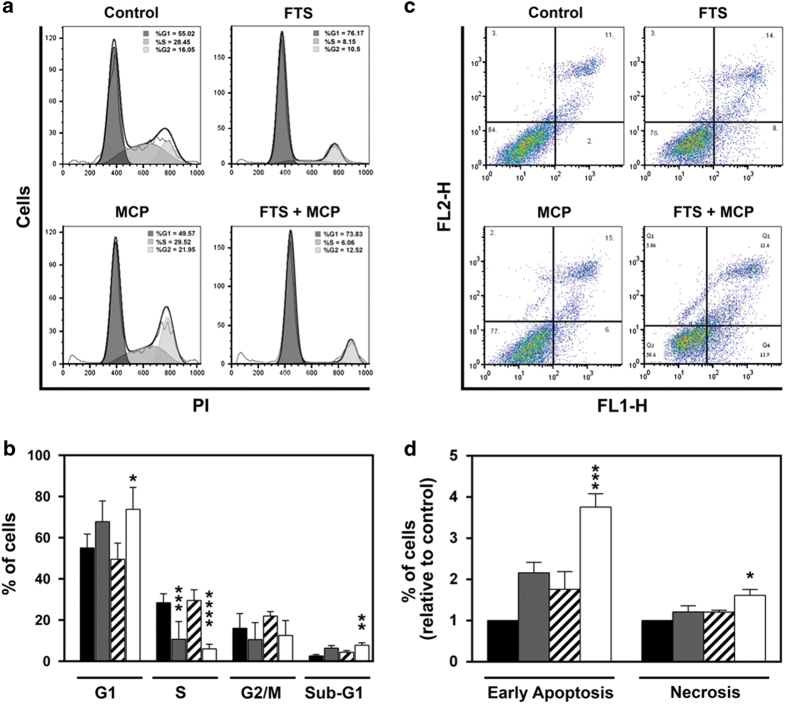
Combined treatment with FTS and MCP induce apoptosis and G1 cell cycle arrest. (**a** and **b**) For cell cycle analysis, ARO cells (5×10^4^) were plated and treated for 72 h either with FTS, MCP, or FTS+MCP. (**a**) Representative histograms of FACS analysis of treated cells by PI staining. (**b**) Quantification of the FACS analysis: amount of cells in G1, S and G2M and sub-G1 cell cycle phases of control (black bar), FTS-treated (gray bar), MCP-treated (upward diagonal bar) and FTS+MCP-treated ARO cells (white bar) are shown. Amount of cells in phases G1 and sub-G1 were significantly higher in FTS+MCP-treated cells compared with untreated cells. Amount of cells in phases S was significantly lower in FTS-treated cells, and FTS+MCP-treated cells compared with untreated cells (**c** and **d**) To examine whether combined treatment of FTS and MCP induces apoptosis, ARO cells (2.5×10^5^) were treated either with FTS, MCP, or FTS+MCP for 48 h. (**c**) Representative dot plot of FACS analysis of the treated cells by Annexin-V and PI staining. Numbers in the quadrants represent the percentage of ARO cells within each quadrant. (**d**) Quantification of the FACS analysis: Left side bars: Apoptotic ARO cells (high Annexin V, low PI) in untreated cells (control-black bar), FTS-treated (grey bar), MCP-treated (upward diagonal bar) and FTS+MCP treated ARO cells (white bar). Right side bars: Necrotic ARO cells (high Annexin V, high PI) in untreated cells (control-black bar), FTS-treated (grey bar), MCP-treated (upward diagonal bar) and FTS+MCP treated ARO cells (white bar). Apoptosis was significantly increased in cells treated with FTS+MCP compared with control. Results are presented as means±S.E.M.; one-way ANOVA analysis revealed significant differences between Annexin-V and PI staining; *post hoc* analysis was performed by the HSD test; *P<0.05, **P<0.01, ***P<0.001, ****P<0.0001. (**a** and **b**: *n*=5; **c** and **d**: *n*=3); FTS, S-farnesylthiosalicylic acid; MCP, modified citrus pectin; PI, phosphatidylinositide.

Having demonstrated that combined treatment with FTS and MCP increased p21 expression, we next examined whether a combined treatment with FTS and MCP affects the cell cycle. To assess the cell cycle, we performed propidium iodide (PI) staining, a well-known method for cell cycle analysis. A representative dot plot of fluorescence-activated cell sorter (FACS) analysis of the treated cell by PI staining is shown in Figure 3a. FTS+MCP significantly increased the percentage of ARO cells in the G1 phase compared with untreated ARO cells (Figure 3b; mean±S.E.M., 76.2±12.1, 73.8±10.7, and 55±6.8, respectively; *P*<0.001, one-way ANOVA; *P*<0.05, Tukey's HSD test). Percentage of cells in phases S was significantly lower in FTS-treated cells and FTS+MCP-treated cells compared with untreated cells (Figure 3b; mean±S.E.M., 10.7±8.6, 6.1±2.2, and 28.5±4.3, respectively; *P*<0.0001, one-way ANOVA; *P*<0.001, Tukey's HSD test). The data suggest that FTS+MCP significantly increased the percentage of ARO cells arrested in the sub-G1 phase as compared with untreated cells (Figure 3b; mean±S.E.M., 7.8±2.6, and 2.6±1.5, respectively; *P*<0.01, one-way ANOVA; *P*<0.01, Tukey's HSD test). In light of these results, we examined whether FTS+MCP induce apoptosis in ARO cells. Apoptosis analysis was performed by the Annexin-V and PI staining methodology.^31^ Representative dot plot results of FACS analysis are shown in Figure 3c. ARO cells that were treated with FTS+MCP had a significantly higher percentage of apoptotic cells compared with untreated control cells (Figure 3d; *P*<0.01, one-way ANOVA; *P*<0.001, Tukey's HSD test). Percentage of necrotic ARO cells was significantly higher in FTS+MCP-treated cells as compared with the control (Figure 3d; *P*<0.05, one-way ANOVA; *P*<0.05, Tukey's HSD test). Our findings indicate that combined treatment with FTS and MCP induces G1 arrest and apoptosis in ARO cells.

### Combined treatment with FTS and MCP inhibits tumor growth *in vivo*

**Figure 4:**
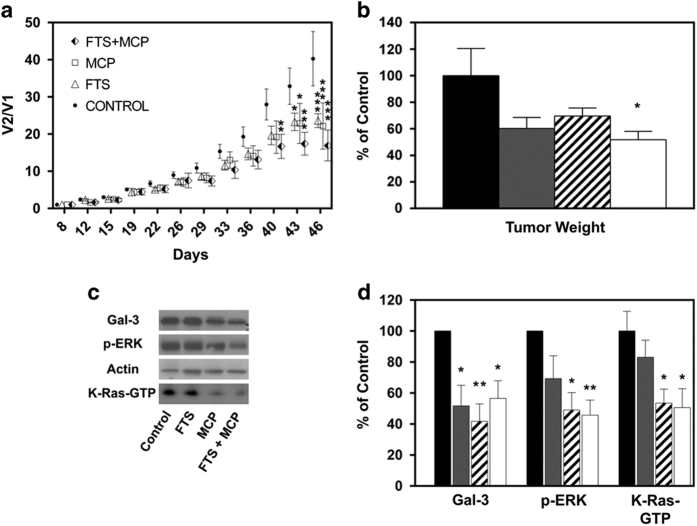
Combined treatment with FTS and MCP inhibit ARO thyroid cell tumor growth in a nude mouse model. ARO cells were injected subcutaneously into the flank areas of nude mice; tumor volumes and weights were determined as described in Materials and Methods. (**a**) Tumor volume in FTS, MCP, FTS+MCP-treated and control mice as a function of time; points—mean; bars—S.E.M.; C—control, F-FTS, M-MCP, F+M-FTS+MCP. (**b–d**) After 35 days, mice were killed and tumors were weighed and then homogenized for immunoblot analysis. (**b**) Tumor weight of control (black bar), FTS (gray bar), MCP (upward diagonal bar) and FTS+MCP (white bar)-treated mice. (**c**) Representative Immunoblots of p-ERK and Gal-3 normalized to actin and K-Ras-GTP, visualized by ECL. (**d**) Apparent levels of Galectin-3, p-ERK, and K-Ras-GTP determined by densitometry (a.u.) of the immunoblots. Results are presented as means±SEM; one-way ANOVA analysis revealed significant differences between tumor volumes, tumor weights and protein levels; *post hoc* analysis was performed by the HSD test and Fisher's LSD test. *P<0.05, **P<0.01, ***P<0.001; FTS, S-farnesylthiosalicylic acid; MCP, modified citrus pectin.

Next, we examined whether combined treatment with FTS and MCP can inhibit the growth of thyroid carcinomas in a mouse model. Mice were injected with ARO cells and treated with FTS, MCP, FTS+MCP or vehicle as described in Materials and Methods. As shown in Figures 4a and b, FTS+MCP significantly decreased tumor volume and tumor weight compared with FTS, MCP and control groups (*P*<0.05, one-way ANOVA; *P*<0.01, Tukey's HSD test). Analysis for the effects of group and time by univariate two-way ANOVA revealed a significant effect of group and time (*P*<0.0001). There was a significant effect for the interaction of group×time (*P*<0.01). The results of pharmacodynamics analysis are shown in Figures 4c and d. Gal-3 and p-ERK levels were significantly decreased by FTS+MCP treatment (*P*<0.05, one-way ANOVA; *P*<0.05, Fisher least significant difference (LSD) test). In addition, FTS+MCP treatment significantly reduced K-Ras-GTP expression levels (Figures 4c and d; *P*<0.05, one-way ANOVA; *P*<0.05, Tukey's HSD test). Taken together, these results show that combined that FTS and MCP hit their targets in the tumors and inhibit the growth of anaplastic thyroid tumor *in vivo*.

## Discussion

Anaplastic Thyroid carcinoma is an extremely aggressive solid tumor that resists most treatments and is almost always fatal.^3,32^ Gal-3 serves as a diagnostic marker to differentiate between benign and malignant human thyroid cancers,^33^ and the role of Ras in these tumors is also well documented.^23^ As Gal-3 was reported to act as a specific binding partner of activated K-Ras and that this interaction promotes K-Ras activation,^5^ we addressed the possible usage of these targets for a possible therapeutic modality against anaplastic thyroid carcinoma. We report here that combined treatment with FTS and MCP inhibited ARO cells proliferation *in vitro* and reduced tumor growth *in vivo*. FTS is a potent Ras inhibitor, which interferes with the interactions between active Ras (Ras-GTP) and the cell membrane, mostly by disrupting the interactions between galectin and Ras-GTP in the cellular plasma membrane. Dislocation of Ras from the plasma membrane to the cytoplasm leads to its degradation.^5,12,34,35^ FTS has been shown to inhibit tumor growth in several types of cancer.^21,23,36,37^ MCP is a specific gal-3 inhibitor that can bind the carbohydrate-binding domain of Gal-3, due to its sugar groups, and block its activity. Previous studies have found that MCP inhibits tumor growth, angiogenesis, and spontaneous metastasis of colon carcinoma and breast cancer.^25,26,38,39^

Thyroid cancer cells express Gal-3,^23,33,40,41^ a beta-galactoside-binding lectin, which associates with the development and malignancy of many types of tumors.^42^ Overexpression of Gal-3 promotes neoplastic transformation by interacting with K-Ras-GTP, enhancing K-Ras-GTP anchorage to the cell membrane, and enabling it to activate downstream signal pathways.^5^ In the present study, we found that combined treatment with FTS and MCP significantly decreased K-Ras and Gal-3 expression. Low K-Ras and Gal-3 levels were associated with inhibition of tumor cells growth *in vitro* and *in vivo*.^23^ The results presented here are in line with a previous study showing that FTS inhibits thyroid cancer cells proliferation *in vitro* and *in vivo*, reduces the expression levels of Ras-GTP and its downstream signaling molecule p-ERK, and increases the expression levels of p21.^23^

P21 is an important cell cycle regulator, which induces cell cycle arrest at G1 and G2/M phases.^28,43^ Indeed, our results showed that combined treatment with FTS and MCP increased p21 levels and induced cell cycle arrest at the G1 phase. P21 is negatively regulated, in part, by Ras and, accordingly, FTS increases p21 levels.^23,29^ Furthermore, p21 levels are upregulated in Gal-3 knockdown PC3 cells (human prostate cancer) along with cell cycle arrest at the G1 phase.^38^ Gal-3 promotes cell cycle progression by enhancing the expression of cyclin D and c-MYC.^44–46^ In myeloma cells, MCP induces G1 arrest and apoptosis via upregulating p21 expression.^39^ Our results showed that combined treatment with FTS+MCP significantly decreased p53 levels. P53 is an important tumor suppressor gene that binds to the DNA and activates hundreds of genes including p21.^47^ However, p21 expression may be regulated independently of p53.^47^ Conversely, as ARO cells express high levels of mutant p53 protein,^48^ suggesting that the reduction in the level of p53 protein expression might imply a positive outcome. We also report that a combined treatment of FTS and MCP inhibitors resulted in apoptosis of ARO cells. It was previously shown that MCP induces apoptosis in multiple myeloma cells and triggers apoptosis associated with activation of caspase-8 and caspase-3 pathway followed by proteolytic cleavage of poly (ADP-ribose) polymerase PARP.^25^

The present results in the preclinical- a nude mouse model system showed the feasibility of this suggestion because FTS and MCP reduced the expression levels of Gal-3, active K-Ras-GTP and phospho-ERK in ARO tumors and significantly reduced tumor size. Taken together, the results depicted here support the suggestion that K-Ras and Gal-3 inhibition should be considered as a novel therapeutic treatment for patients suffering from anaplastic thyroid carcinoma.

## Materials and Methods

### Cell culture and reagents

ARO cells were maintained in RPMI-1640 medium (Biological Industries, Kibbutz Beit Haemek, Israel) supplemented with 10% fetal calf serum, 100 units/ml penicillin, and 5% L-glutamine. All reagents were purchased from Biological Industries. Cells were incubated at 37 °C in a humidified atmosphere of 95% air/5% CO_2_. FTS was kindly donated by Concordia Pharmaceuticals (Fort Lauderdale, FL, USA). MCP was prepared as described.^26^

### Proliferation and cell survival assay

For the FTS dose–response experiment, ARO cells were plated in a 96-well plate (0.5×104 cells/well); after 24 h, cells were treated with FTS at different concentrations (25, 37, 50, 62.5, 75 and 100 *μ*M) or, as a control, with 0.1% dimethyl sulfoxide for 96 h. Cell viability was assessed by the methylene blue staining assay. For the MCP dose–response experiment, ARO cells were plated (0.5×10^5^ cells/well) in a 24-well plate; after 24 h, cells were treated with MCP at different concentrations (0.1, 0.2, 0.3, 0.4, and 0.5%) or, as a control, with 0.5% D-lactose. After 96 h, cells were counted. To measure the combined effect of FTS and MCP, ARO cells were plated in a 24-well plate (0.5**×**10^5^ cells/well); after 24 h, cells were treated either with FTS in different concentrations (25, 50, 75 and 100 *μ*M) plus the IC_50_ of MCP or with MCP in different concentrations (0.1, 0.2, 0.3 and 0.4%) plus the IC_50_ of FTS. After 96 h, cells were counted. Cell viability was calculated as the ratio of live cells in treated cultures to that in untreated cultures.

### Methylene blue staining assay

Cells were fixed with 4% formaldehyde in phosphate-buffered saline (PBS) for 2 h, then washed once with 0.1 M boric acid (pH 8.5) and incubated with the DNA-binding dye methylene blue (1% in boric acid) for 20 min at room temperature. Cells were then washed three times with distilled water and lysed with 0.1 M HCl. Absorbance was measured with a Tecan Spectrafluor Plus spectrophotometer (Mannedorf, Switzerland) at 595 nm. Cell viability was calculated as the ratio of absorbance in treated cultures to that in untreated control cultures.

### Immunoblot analysis

ARO cells were plated in 10-cm plates (8x10^5^ cells) and grown for 24 h. Cells were then treated either with 0.35% MCP, 75 *μ*M FTS, 0.35% MCP plus 75 *μ*M FTS or as a control with 0.35% D-lactose for 48 h. In a second set of experiments, cells were treated either with 0.35% MCP, 75 *μ*M FTS, 0.35% MCP plus 75 *μ*M FTS or as a control with 0.35% D-lactose for 72 h. Next, cells were lysed in 300 *μ*l homogenization buffer (50 mmol/l Tris-HCl—pH 7.6, 20 mM MgCl_2_, 200 mM NaCl, 0.5% NP40, 1 mM DTT, and protease inhibitors), centrifuged for 10 min at 14 000 r.p.m. at 4 °C and the supernatant was collected. Equal amounts of proteins (40 *μ*g per lane) were subjected to SDS-PAGE, followed by immunoblotting with mouse anti-pan-Ras antibody (Ab, Calbiochem, San Diego, CA, USA), rabbit anti-*β* tubulin Ab (Sigma Aldrich, Rehovot, IL, USA), mouse anti- K-Ras (Calbiochem), rat anti-Galectin-3 (Mac2), rabbit anti-total ERK (Santa Cruz, Dallas, TX, USA), mouse anti-p-ERK (Sigma Aldrich), rabbit anti-p21 (Santa Cruz), mouse anti-p53 (Calbiochem) and mouse anti-actin (MP Biomedicals, Santa Ana, CA, USA). Blots were then exposed to the appropriate secondary peroxidase-coupled IgG (Jackson ImmunoResearch Laboratories, West Grove, PA, USA) and subjected to enhanced chemiluminescence. Protein bands were quantified by densitometry with Image EZQuant-Gel software (EZQuant Ltd, Tel-Aviv, Israel).

### GTPase pull-down assays

To measure Ras-GTP levels, we used the GTPase pull-down assays. Lysates containing 1 mg protein were used to determine the Ras-GTP content by the glutathione *S*-transferase-RBD (Ras-binding domain of Raf) pull-down assay, followed by western immunoblotting with pan-Ras Ab and K-Ras Ab as described elsewhere.^35^

### Cell cycle analysis

To test whether FTS and MCP affect cell cycle, ARO cells were plated in six-well plates (5×10^4^ cells per well), and treated 24 h later either with FTS (75 *μ*M), MCP (0.35%), MCP plus FTS or, as a control, with D-lactose (0.35%) for 48 h. After 48 h, cells were collected, pelleted, washed with PBS, resuspended in 0.5 ml PBS and treated with RNase A/T1 (0.2%, Thermo Scientific, Waltham, MA, USA) to remove RNAs from cells (30 min, 37 °C). Then, 0.05% PI (50 *μ*g/ml, Sigma Aldrich) and 0.05% Triton X-100 (1%, Sigma Aldrich), were added to the cells. Flow cytometry analysis was performed using Becton Dickinson FACSort (Los Angeles, CA, USA), and the results were analyzed with FlowJo software (Ashland, OR, USA). All experiments were carried out in duplicate and performed five times.

### Apoptosis assay

To quantify the percentage of cells undergoing apoptosis, we used Annexin-V–FITC (MEBCYTO Apoptosis kit, MBL, Nagoya, Japan). ARO cells were plated in six-well plates (2×10^5^ cells per well), and treated 24 h later either with FTS (75 *μ*M), MCP (0.35%), MCP plus FTS or as a control with D-lactose (0.35%) for 48 h. After 48 h, cells were collected, washed with PBS, resuspended in binding buffer and assayed by double staining with Annexin-V–FITS and PI according to the manufacturer’s instructions. Flow cytometry analysis was performed using Becton Dickinson FACSort and the results were analyzed with FlowJo software. All experiments were carried out in duplicate and performed three times.

### *In vivo* experiment

Athymic nude mice (6 weeks old) were obtained from Harlan Laboratories Limited (Jerusalem, Israel). Mice were kept at the Life Sciences Faculty, Tel Aviv University, animal facility, under standard conditions, 23±1 °C, 12 h light cycle (0700±1900 hours) with *ad libitum* access to food and drink. On day 0, ARO cells (2.5×10^6^ in 0.1 ml of PBS) were implanted subcutaneous, just above the right femoral joint. FTS (40 mg/kg) was given daily by oral administration with 0.1 ml CMC (0.5% w/v). MCP (0.5%) was given in mice drinking water (5 ml/day). When tumor volumes reached 0.3 to 0.5 cm^3^, mice were randomly separated into four groups: control, FTS, MCP, and FTS+MCP. Control mice were fed daily with 0.1 ml CMC and received CP (0.5%) in their drinking water (5 ml/day); the FTS group received 0.5% CP in their drinking water (5 ml/day). MCP mice were fed daily with 0.1 ml CMC. Tumor volumes were measured every 4 days as previously described.^35^ After 35 days, mice were killed and tumors were weighed and then homogenized for immunoblot analysis in lysis buffer (10% w/v). The Tel Aviv University Animal Welfare Committee approved all procedures.

### Statistical analysis

Results are expressed as mean values±S.E.M. *P*-values were calculated by one-way ANOVA and two-way ANOVA. *Post hoc* analysis was performed by Tukey's HSD test and by Fisher's LSD test.

## Abbreviations

CI: combination index
CP: citrus pectin
FACS: Fluorescence-activated cell sorter
FTS: S-trans, transfarnesylthiosalicylic acid
Gal-3: Galectin-3
HSD: honest significant difference
IC_50_: half-maximal inhibitory concentration
LSD: least significant difference
MCP: modified citrus pectin
PI: propidium iodide.
